# Electronic self-monitoring of mood using IT platforms in adult patients with bipolar disorder: A systematic review of the validity and evidence

**DOI:** 10.1186/s12888-016-0713-0

**Published:** 2016-01-15

**Authors:** Maria Faurholt-Jepsen, Klaus Munkholm, Mads Frost, Jakob E. Bardram, Lars Vedel Kessing

**Affiliations:** Psychiatric Center Copenhagen, Rigshospitalet, Blegdamsvej 9, Copenhagen, DK- 2100 Denmark; The Pervasive Interaction Laboratory (PIT Lab), IT University of Copenhagen, Copenhagen, Denmark; DTU Compute Copenhagen Center for Health Technology, DTU, Lymgby, Denmark

**Keywords:** Bipolar disorder, Electronic self-monitored mood, Validity, Evidence, Systematic review, Depressive and manic symptoms

## Abstract

**Background:**

Various paper-based mood charting instruments are used in the monitoring of symptoms in bipolar disorder. During recent years an increasing number of electronic self-monitoring tools have been developed. The objectives of this systematic review were 1) to evaluate the validity of electronic self-monitoring tools as a method of evaluating mood compared to clinical rating scales for depression and mania and 2) to investigate the effect of electronic self-monitoring tools on clinically relevant outcomes in bipolar disorder.

**Methods:**

A systematic review of the scientific literature, reported according to the Preferred Reporting items for Systematic Reviews and Meta-Analysis (PRISMA) guidelines was conducted. MEDLINE, Embase, PsycINFO and The Cochrane Library were searched and supplemented by hand search of reference lists. Databases were searched for 1) studies on electronic self-monitoring tools in patients with bipolar disorder reporting on validity of electronically self-reported mood ratings compared to clinical rating scales for depression and mania and 2) randomized controlled trials (RCT) evaluating electronic mood self-monitoring tools in patients with bipolar disorder.

**Results:**

A total of 13 published articles were included. Seven articles were RCTs and six were longitudinal studies. Electronic self-monitoring of mood was considered valid compared to clinical rating scales for depression in six out of six studies, and in two out of seven studies compared to clinical rating scales for mania.

The included RCTs primarily investigated the effect of heterogeneous electronically delivered interventions; none of the RCTs investigated the sole effect of electronic mood self-monitoring tools. Methodological issues with risk of bias at different levels limited the evidence in the majority of studies.

**Conclusions:**

Electronic self-monitoring of mood in depression appears to be a valid measure of mood in contrast to self-monitoring of mood in mania. There are yet few studies on the effect of electronic self-monitoring of mood in bipolar disorder. The evidence of electronic self-monitoring is limited by methodological issues and by a lack of RCTs. Although the idea of electronic self-monitoring of mood seems appealing, studies using rigorous methodology investigating the beneficial as well as possible harmful effects of electronic self-monitoring are needed.

**Electronic supplementary material:**

The online version of this article (doi:10.1186/s12888-016-0713-0) contains supplementary material, which is available to authorized users.

## Background

In bipolar disorder research, there has during the last decade been an emerging shift in illness paradigm from a focus on affective episodes to an increasing focus on inter-episodic mood instability [1, 2]. Many patients with bipolar disorder remain symptomatic during inter-episode periods and experience significant subsyndromal day-to-day or week-to-week mood swings that are of greater severity than those experienced by healthy individuals and appear to reflect illness activity [2]. Further, these subsyndromal mood swings seem associated with high risk of relapse, hospitalization and impaired functioning [1, 3–5]. Continuous monitoring and assessment of mood instability and other variables possibly reflecting illness activity in detail, including measures of duration, severity and frequency of symptoms, may therefore be clinically relevant since it would allow for early intervention on subsyndromal symptoms and ultimately prevention of full-blown affective episodes. Self-reports are ubiquitous in psychiatric research, and various mood charting instruments for self-monitoring are frequently used in the management and monitoring of depressive and manic symptoms in patients with bipolar disorder. Traditionally these mood charting instruments have been paper-based, such as the National Institute of Mental Health LifeChart Method (NIMH-LCM) [6], the Systematic Treatment Enhancement Program for Bipolar Disorder (STEP-BP) the Mood Chart (mood chart no longer available online) and the ChronoSheet [7] and have been shown valid compared to clinical rating scales for depression and mania [8, 9]. Paper-based mood charting instruments can be viewed as facilitating tools helping patients with bipolar disorder gain illness insight, facilitate patient empowerment, teach patients to recognize early warning signs of recurrence of affective episodes and enable individualized characterization of mood instability in detail. However, several issues limiting the usefulness of paper-based mood charting instruments have been addressed, such as low compliance and potential recall bias when reporting data retrospectively, i.e. where patients complete batches of daily ratings at a single time (sometimes referred to as *hoarding* or *backfilling*) [10–13]. During recent years there has been an increasing growth of e-mental health technologies [14], including electronic platforms offering tools for self-monitoring of mood. The electronic approach for self-monitoring of mood offers ecological momentary assessments [15], a monitoring technique for assessment in real-time and in naturalistic settings, offers the ability to verify the timing and compliance of data collection, eliminates the need for costly and error-prone data entry, may help remind patients to perform the self-monitoring and may have higher usability than paper-based versions.

However, it remains unclear whether the severity of self-monitored mood registered using electronic self-monitoring tools is a valid measure compared to validated clinical rating scales for depression and mania, which are currently used as the golden standard for assessing the severity of depressive and manic symptoms in patients with bipolar disorder. Furthermore, it remains unclear to what extent the use of electronic mood self-monitoring tools affects clinically relevant outcomes, and importantly whether there may in fact, be harmful effects, e.g. self-monitoring of mood symptoms may induce depressive ruminations that may result in increasing severity of depressive symptoms [16]. An understanding and overview of these aspects is crucial in order to guide the use and development of IT platforms for electronic self-monitoring of mood in bipolar disorder.

The objectives of the present systematic review were thus 1) to evaluate the validity of electronic mood self-monitoring tools compared to validated clinical rating scales for depression and mania and 2) to evaluate the evidence of the effect of electronic mood self-monitoring tools on clinically relevant outcomes in randomized controlled trials (RCT).

This is the first systematic review of electronic self-monitoring of mood in patients with bipolar disorder.

## Methods

This systematic review was conducted and reported according to the Preferred Reporting Items for Systematic Reviews and Meta-Analysis (PRISMA) statement [17]. Methods of the review and eligibility criteria were established in advance and documented in a review protocol that can be retrieved from the authors upon request. No modifications were made to the review protocol during the review process.

### Study selection

#### Eligibility criteria

Original studies involving IT platforms for electronic self-monitoring of mood used by adult patients with bipolar disorder ≥18 years of age and either reporting on correlations between electronically self-monitored mood and validated clinical rating scales for depression and mania or RCTs assessing the effect of electronic self-monitoring tools as an intervention were eligible for review. The language of the publications was restricted to English. The types of IT platforms for electronic self-monitoring of mood were defined as cell phones, computers, tablets, PDAs, smartphones and online devices. Papers only describing the technical part of the IT platforms, not including patients with bipolar disorder and/or not reporting on correlations between electronic mood self-monitoring and validated clinical rating scales for depression and mania or the effect of electronic self-monitoring as an intervention in RCTs in patients with bipolar disorder were excluded from review. Where multiple articles reported on different validated clinical rating scales deriving from the same study and reported on the same patient sample, all of these articles were included for review.

#### Information sources and search strategy

Published studies were identified through searching the electronic databases MEDLINE (January 1950 to July 2015), PsychINFO (1806 to July 2015), Embase (1974 to July 2015) and The Cochrane Library (issue 6, 2015) supplemented by hand search of reference lists of retrieved articles.

The literature search was conducted by one researcher (MFJ) and employed the search terms/key words: (telephone or mobile phone or cellular phone or cell phone or smartphone or computer or telecommunications or electronic or electronic device or text message or application or app) and (bipolar disorder or manic depressive psychosis or manic depressive disorder or mania or bipolar affective disorder or manic or bipolar depression).

#### Study selection and data extraction

**Fig. 1:**
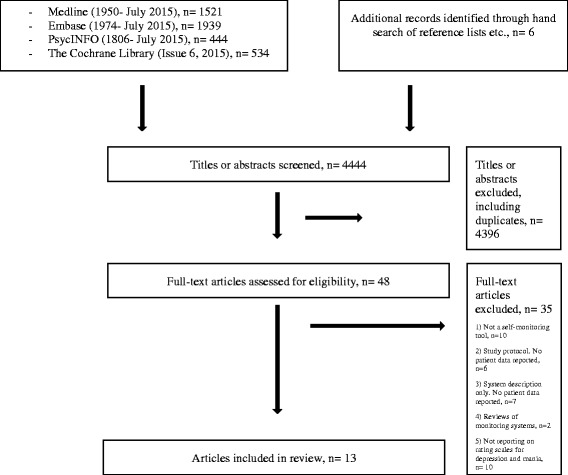
Flow diagram of literature search and study selection process

The flow diagram of the study selection process is presented in Fig. 1. All retrieved titles and abstracts were screened for eligibility by one researcher (MFJ). All potentially relevant articles were retrieved and full-text articles were then assessed for fulfilling eligibility. One reviewer (MFJ) extracted the data from the included articles and a second reviewer (KM) independently checked the extracted data. Any disagreements between the researchers were resolved by discussion between three of the researchers (MFJ, KM and LVK). The risk and types of bias assessed in individual studies were selection bias, performance bias, detection bias, attrition bias, reporting bias and other biases as suggested by The Cochrane Collaboration [18]. Any disagreements or uncertainties related to bias evaluation were resolved by discussion between the authors.

#### Ethics

The present systematic review did not need ethical approval. Data presented are from individual studies approved by local ethical commitees and consent obtained in individual studies.

## Results

### Study selection

The results of the literature search and selection of studies are presented in Fig. 1. The literature search involving the MEDLINE, PsychInfo, Embase and The Cochrane Library databases identified a total of 4438 titles and additionally six titles were identified by hand search. Of these, 4396 titles, including duplicates, were excluded from review due to not fulfilling the eligibility criteria, the main reasons for exclusion being 1) not involving self-monitoring 2) not including patients with bipolar disorder and 3) only providing the technical description of the IT platforms. Thus, 48 full-text articles were further evaluated for eligibility. Of these, 35 articles were excluded from review due to 1) not describing a mood self-monitoring tool (*n* = 10) [19–28] 2) not reporting on patient data (*n* = 13) [29–41], 3) being review articles (*n* = 2) [42, 43], and 4) not reporting on validated clinical rating scales for depression and mania (*n* = 10) [44–53]. A total of 13 articles fulfilled the eligibility criteria and were included in the qualitative analysis [12, 16, 54–64].

### Study characteristics

Of the 13 included articles seven were RCTs [16, 57, 58, 60–63] and six had a longitudinal design (non-RCTs) [12, 54–56, 59, 64]. One article did not present data on follow-up period [55]. The remaining articles reported follow-up periods ranging from 2 weeks to 24 months. The sample sizes ranged from 10 to 233 patients and the mean age ranged from 33.4 to 47.5 years. The included studies were conducted in Europe [16, 55, 59, 60, 64], USA [12, 56–58, 62], Canada [54], and Australia [61, 63], respectively. The different IT platforms used for electronic self-monitoring of mood in the studies were computers (using e-mail or web interfaces) [12, 54, 55, 57, 60, 61, 63, 64], PDAs [56, 64] and smartphones [16, 58, 59, 62, 64], respectively.

### Validity of electronic self-monitored mood compared to validated clinical rating scales for depression and mania

Seven articles evaluated the validity of different electronic mood self-monitoring tools compared to validated clinical rating scales for depression and mania [12, 54–56, 58, 59, 64] (Table 1). The studies included a total of 206 patients with bipolar disorder with a self-monitoring period ranging from 2 weeks to 18 months. Four out of the seven articles reported on compliance to mood self-monitoring with a mean level missing data ranging between 6.1–57.9 %. Three studies did not provide information on this matter.

**Table 1:** Characteristics of studies evaluating the validity of electronic self-monitoring tools of mood compared to validated clinically administrated rating scales for depression and mania in patients with bipolar disorder listed according to year of publication. *N* = 206

Author, Country, year of publication	Design	IT platform, name of tool	Sample size, setting, age, bipolar type I	Self-monitoring frequency, follow-up	Blinding of outcome assessor	Rating scale	Corresponding days between self-monitoring data and ratings in analyses	Correlation analyses	Missing self-monitored data
Whybrow PC et al., USA, Canada & Germany, 2003 [12]	Longitudinal	Computer, ChronoRecord	80, Outpatient, 38.67 (SD 10.86), 72.5 %	Daily, 3 months	NA	HDRS^a^,YMRS^b^	Yes	*Pearson:* HDRS: *r* = −0.683 (*p* < 0.001)	6.1 % (SD 9.3)
								YMRS non-significant	
Bauer M et al., USA, Canada & Germany, 2004 [54]	Longitudinal	Computer, ChronoRecord	80, Outpatient, 38.67 (SD 10.86), 72.5 %	Daily, 3 months	NA	HDRS,YMRS	Yes	*Pearson:* HDRS: *r* = −0.683 (*p* < 0.001)	6.1 % (SD 9.3)
								YMRS non-significant	
								*Linear mixed model coefficient:* HDRS: β = −0.303 (*p* < 0.001)	
								YMRS non-significant	
Bauer M et al., USA, Canada & Germany, 2008 [55]	Longitudinal	Computer, ChronoRecord	27 Inpatient & 80 Outpatient,	Daily, NA	Yes	YMRS, MRS ^c^	Yes	*Pearson:* YMRS: *r* = 0.825 (*p* < 0.001)	NA
								*Linear mixed model coefficient:* YMRS: β = 0.229 (*p* = 0.001)	
Depp CA et al., USA, 2010 [56]	Longitudinal	PDA, PRISM	10, Outpatient, 41.0 (SD 13.7)	Daily, 2 weeks	NA	MADRS^d^, YMRS	NA	*Pearson:* MADRS: *r* = 0.841 (*p* = 0.018)	22 % (SD 14)
								YMRS non-significant	
Depp CA et al., USA, 2012 [58]	RCT	Self-monitoring using smartphone	18, Outpatient, 44.0 (SD 14), 90.9 %	Twice/ day in fixed time blocks, 12 weeks	Yes	MADRS^d^, YMRS	Yes	*Pearson:* Baseline: MADRS: *r* = −0.567 (*p* = 0.014)	57.9 % (SD 26.6)
								YMRS non-significant	
								6 weeks: MADRS: *r* = −0.542 (*p* = 0.028)	
								YMRS: *r* = 0.520 (*p* = 0.032)	
Faurholt-Jepsen M et al., Denmark, 2014 [59]	Longitudinal	Smartphone, MONARCA	17, Outpatient, 33.4 (SD 9.5), 82.4 %	Daily, 3 months	Yes	HDRS, YMRS	Yes	*Linear mixed model coefficient:* HRDS: β = −0.051 (*p* < 0.001)	NA
								YMRS non-significant	
Schärer LO et al., Germany, 2015 [64]	Longitudinal	Computer, PDA, smartphone, Personal Life-Chart app	54, Outpatient, 40.6	Daily, 18 months	Yes	IDS-C^e^, YMRS	Yes	*Spearman correlation coefficient:* IDS-C: *r* = 0.60–0.62 (*p*-value not shown)	NA
								YMRS: *r* = 0.17–0.39 (*p*-value not shown)	

^a^Hamilton Depression Rating Scale

^b^Young Mania Rating Scale

^c^Bech-Rafaelsen Manic Rating Scale

^d^Montgomery Asberg Depression Rating Scale

^e^The Inventory of Depressive Symptomatology, clinician-rated

#### Correlation with clinically rated depressive symptoms

Six (*n* = 179) of the seven articles evaluated the validity of electronic self-monitoring of mood compared to clinical rating scales for depression using the Montgomery Asberg Depression Rating Scale (MADRS) [65], the Hamilton Depression Rating Scale (HDRS) [66] or the Inventory of Depressive Symptomatology, clinician rated (IDS-C) [67]. All of the six articles found a significant correlation between self-monitored mood and clinical rating scales for depression, indicating that increasing severity of self-monitored depressive mood correlated with increasing scores of clinical rating scales for depression. Regression coefficients and slopes are presented in Table 1, but not all studies provided both of these statistical measures.

The monitoring tools used were PDAs [56, 64], computers [12, 54, 64] and smartphones [58, 59, 68], respectively. All but one article, which did not provide data on this in the paper [56] investigated the validity of electronic self-monitoring compared to clinical rating scales for depression from corresponding days.

#### Correlation with clinically rated manic symptoms

All of the seven articles evaluated the validity of electronic self-monitoring of mood compared to clinical rating scales for mania using the Young Mania Rating Scale (YMRS) [69]. Two articles (*n* = 64) found a significant correlation between self-monitored mood and the clinical rating scale for mania [55, 64], indicating that increasing severity of self-monitored manic mood correlated increasing scores on the clinical rating scale for mania. One study (*n* = 18) found a significant positive correlation between self-monitored mood and the clinical rating scale for mania after 6 weeks of mood self-monitoring, but not at baseline [58]. Regression coefficients and slopes are presented in Table 1, but not all studies provided both of these statistical measures. The monitoring tools used were PDAs [56, 64], computers [12, 54, 55, 64] and smartphones [58, 59, 64], respectively. All but one article, which did not provide data on this in the paper [56] investigated the validity of electronic self-monitoring compared to clinical rating scales for mania from corresponding days.

### Effect of electronic mood self-monitoring tools on clinically relevant outcomes in randomized controlled trials

Of the 13 included articles seven were RCTs including a total of 759 patients with bipolar disorder involving a follow-up period ranging from 12 weeks to 12 months [16, 57, 58, 60–63] (further details are described in Table 2 and in Additional file 1).

**Table 2:** Characteristics of studies on randomized controlled trials (RCT) investigating the effect of IT platforms with intervention programmes including electronic self-monitoring of mood on different outcomes in adult patients with bipolar disorder listed according to year of publication. *N* = 759

Author, Country, year of publication	Design	Intervention group: IT platform, name of tool	Control group	Sample size, setting, age^a^, Bipolar disorder type I	Additional information on the intervention group	Self-monitoring frequency, follow-up	Blinding of outcome assessor	Outcome
Lieberman DZ et al., USA, 2010 [57]	RCT	Computer-based, Online self-monitoring using LCM^b^	Paper-and-pencil self-monitoring	48, Outpatient, 37.7 (SD 12.5), 13 %	Online self-monitoring of mood, medications and other variables	Daily, 90 days	NA	- No difference in CGI-S^c^ score between the groups
								- Significantly higher number of days rated and with complete data in the electronic self-monitoring group (intervention group)
Depp CA et al., USA, 2012 [58]	RCT	Smartphone-based, Self-monitoring using smartphone	Paper-and-pencil self-monitoring	40, Outpatient, 45.1 (SD 13.8), 90.4 %	Smartphone-based monitoring of momentary mood and related experiences	Twice/ day in fixed time blocks, 12 weeks	Yes	- Significantly higher variability of self-monitored mood in the electronic self-monitoring group both within-person and between-persons
								- Significantly higher compliance in paper-and pencil group (control group)
Todd NJ et al., UK, 2014 [60]	RCT	Web-based, Online self-monitoring using ‘Living with Bipolar’	TAU (and waiting list)	122, Outpatient, 43.44 (SD 11.25), 70 %	Web-based recovery informed self-management and self-monitoring	NA, 6 months	No (self-assessed unblinded by patients)	- Primary outcome: Significantly higher self-assessed quality of life (QoL.BD-Brief^d^ and WHOQoL-BREF^e^) in the intervention group
								- Secondary outcome: Significantly higher self-assessed recovery (BRQ ^f^), lower symptoms severity (ISS^g^), and higher social functioning (SASS^h^) in the intervention group
Barnes CW et al., Autralia, 2015 [61]	RCT	Web-based, Online self-monitoring using ‘Recovery Road for Bipolar Disorder’	Websites on ‘healthy lifestyles’ (and waiting list)	233, Outpatients, 39.0 (SD 10.8), 87.6 %	Web-based psychoeducatio-nal program and self-monitoring	Weekly, 12 months	No (self-assessed unblinded by patients)	- Primary outcome: No significant differences in self-reported time to recurrence (BDI-II^i^, ISS^g^, self-reported hospitalization and Sheehan Disability Scale) between the groups
Depp CA et al., USA, 2015 [62]	RCT	Smartphone-based, Self-monitoring using ‘Personalized Real-Time Intervention for Stabilizing Mood (PRISM)’	Paper-pencil self-monitoring	82, Outpatient, 47.5 (SD 12.8), 87.8 %	Smartphone-based personal self-management strategies and self-monitoring	Twice/ day in fixed time blocks, 24 weeks	Yes	- Primary outcome: Significantly lower MADRS^j^ score at week 6 and 12 in the intervention group. No difference at end of study.
								- Secondary outcome: No significant differences in YMRS^k^ and self-rated functional impairment^l^ between the groups
Lauder S et al.,Australia, 2015 [63]	RCT	Web-based, Online self-monitoring using ‘Moodswings’	Moodswings (online psychoeducation, self-monitoring of mood and discussion boards)	156, Outpatients, 40.6 (SD 10.6), 51.5 %	Moodswings plus online Cognitive Behavioral Therapy	Daily, 12 months	No (self-assessed unblinded by patients)	- Primary outcome: Significantly lower ASRM^m^ score in the intervention group. No difference in MADRS Self-assessment and other self-assessed outcomes between the groups.
Faurholt-Jepsen M et al., Denmark, 2015 [76]	RCT	Smartphone-based, self-monitoring using ‘MONARCA’	Placebo smartphone and TAU	78, Outpatients, 29.3 (SD 8.43), 67.1 %	Smartphone-based self-monitoring and daily feedback loop to patient and clinicians	Daily, 6 months	Yes	- Primary outcome: No significant difference in HDRS-17^n^ and YMRS between the groups.
								- Sub-analyses: More depressive symptoms in the intervention group. Fewer manic symptoms in the intervention group.

^a^Mean and standard deviation (SD) unless otherwise stated

^b^Life Chart Method

^c^Clinical Global Impression Severity (CGI-S)

^d^Quality of Life in BD scale (Brief version)

^e^World Health Organisation Quality of Life assessment tool, brief version

^f^Bipolar Recovery Questionnaire

^g^Internal States Scale

^h^Social Adaptation Self-evaluation Scale

^i^Beck Depression Inventory-II

^j^Montgomery Asberg depression Rating Scale Self-Assessment

^k^Young Mania Rating Scale

^l^Illness Intrusiveness Scale

^m^Altman Self-Rating Mania Scale

^n^Hamilton Depression Rating Scale 17-item

Two of the RCTs aimed primarily at investigating differences in compliance rates between using an electronic mood self-monitoring tool compared to using paper-and-pencil mood self-monitoring [57, 58] and reported on differences in symptom scores as secondary outcomes. The first study (*n* = 48) reported that the intervention group showed significantly higher compliance to mood self-monitoring compared to the control group. No difference in Clinical Global Impression Severity (CGI-S) score was found between the two groups [57]. The second study (*n* = 40) reported that the intervention group showed significantly higher variability of self-monitored mood compared to the control group. Further, a higher compliance to mood self-monitoring was found in the control group [58].

Five of the seven included RCTs investigated the effect of different electronically delivered intervention programmes including a self-monitoring tool on symptoms of illness activity in patients with bipolar disorder [16, 60–63].

One study (*n* = 122) reported on a RCT investigating the effect of an online interactive intervention based on principles of Cognitive Behavioral Therapy and psychoeducation including self-monitoring (‘Living with Bipolar’) (intervention group) compared to treatment-as-usual (TAU) (control group). The study reported that the intervention group showed significant improvement in self-assessed quality of life, recovery, symptoms severity and social functioning compared to the control group [60].

Another study (*n* = 233) reported on a RCT investigating the effect of a web-based psychoeducational program including self-monitoring (Recovery Road for Bipolar Disorder) (intervention group) compared to a control group directed to web-sites on healthy lifestyles. The study reported that there were no significant differences between the two groups in any of the defined outcome [61].

The third study (*n* = 82) reported on a RCT investigating the effect of smartphone-based personalized self-management strategies based on self-reported mood scores (intervention group) compared to paper-and-pencil mood self-monitoring (control group). The study reported no significant differences in depressive and manic symptoms between the two groups at the end of study [62].

The fourth study (*n* = 156) reported on a RCT investigating the effect of an online psychoeducation, mood tracking, discussion boards and cognitive behavioral therapy program (MoodSwings Plus) (intervention group) compared to an online psychoeducation, mood tracking and discussion boards program (MoodSwings) (control group). The study reported that the intervention group showed significant reductions in self-assessed manic symptoms compared to the control group, but no difference was observed in relation to self-assessed depressive symptoms [63]. The last study, by the authors, (*n* = 78) reported on a RCT investigating the effect of smartphone-based self-monitoring including a feedback loop to the clinicians (MONARCA) (intervention group) compared to a placebo smartphone and TAU (control group). The study reported no significant differences between the two groups in any of the defined outcomes. Sub-analyses showed that the intervention group had more depressive symptoms and fewer manic symptoms than the control group during the study period [16].

#### Risk of bias within individual studies

All of the included articles but one [55] included only outpatients. Notably only one of the longitudinal studies provided data on the patients who were excluded from participating in the study [59]. Three of the six longitudinal studies reported on data collected with assessors who were blinded to the electronic self-monitoring data [55, 58, 59]. Thus, most included longitudinal studies were evaluated to be at risk of selection bias, performance bias and/or detection bias at some level.

In two articles on RCTs no information regarding sequence generation and allocation concealment was provided [57, 58]. Thus, these RCTs were assessed to possibly be at risk of selection bias.

One of the RCTs did not state whether the outcome assessors were blinded to randomization group and were therefore evaluated to be at risk of possible detection bias [57]. Further to this point, given the nature of the intervention (the patients were unblinded to intervention) in the included RCTs none of the studies were double blinded, and therefore naturally all at risk of performance bias. Furthermore, three of the RCTs investigated the effect of different electronically delivered intervention programmes including mood self-monitoring on self-assessed outcome measures with no observer/researcher blinded outcome measures [60, 61, 63].

## Discussion

This is the first systematic review of the evidence of the validity of electronic mood self-monitoring tools using IT platforms as methods for assessing mood in adult patients with bipolar disorder compared with clinical rating scales for depression and mania. Further, the evidence of the effect of electronic mood self-monitoring tools on clinically relevant outcomes in RCTs was assessed. A total of 13 published articles were included. The included articles were heterogeneous, employing various monitoring IT platforms and included different clinically relevant outcomes.

Electronic self-monitored mood was found valid compared to clinical rating scales for depression in six out of six studies comprising a total of 179 outpatients [12, 54, 56, 58, 59, 64], but only two studies found a correlation between electronic self-monitored mood and a validated rating scale for mania [55, 64]. Thus, the present review suggests that despite different bias issues it seems possible for patients to validly evaluate the severity of their depressive symptoms, but specifically difficult to report emerging manic symptoms in a valid way and may be due to decreased illness insight during hypomania/mania [70]. Furthermore, as mentioned most of the studies included only outpatients who did not present with severe levels/ high scores on clinical rating scales for depression and mania. The validity of the electronic self-monitoring tools may be both overestimated as well as underestimated disregarding the possible difficulty of self-monitoring the severity of symptoms in more severe cases.

The studies included describe convergent validity of electronic self-monitoring tools. Other variables such as activity level and sleep length could represent parameters for self-monitoring that may correlate with clinical rating scales for depression and mania, but content validity was not investigated in the present review. Further, the reliability and predictive validity of the electronic self-monitoring tools were not investigated in the present review.

Paper-based self-monitoring tools for depressive mood registered using different types of paper-based tools has been shown to significantly correlate with the scores on clinical rating scales for depression in a number of studies [7, 9, 71–73]. The severity of self-monitored manic mood has been shown to correlate with the scores of clinical rating scales for mania in a number of studies [8, 9, 74, 75], however, not in all studies [7]. Further, other parameters such as activity level may correlate with the scores of clinical rating scales for mania [7]. Two of the RCTs included researcher blinded validated clinical rating scales for depression and mania as outcomes [16, 62]. These studies could potentially investigate the validity of electronic mood self-monitored compared to clinical rating scales for depression and mania and thus further contribute to the knowledge in this area.

It should be mentioned that a paper by the authors analyzing the validity between mood self-monitoring and clinical rating scales for depression and mania has been accepted for publication [76]. This study used a smartphone-based mood self-monitoring tool and found a significant correlation between self-monitored mood and validated ratings scales for both depression and mania, respectively.

Using electronic self-monitoring tools may offer solutions regarding issues of low compliance and potential recall bias that are present when using paper-based self-monitoring tools. However, the results presented in this review suggest that it seems difficult for patients to evaluate manic symptoms calling for other new and more objective real-time electronic methods to monitoring the severity of manic symptoms in patients with bipolar disorder.

To provide a more complete and inclusive picture of the scientific research on electronic self-monitoring the RCTs using electronic self-monitoring tools as a part of an intervention were evaluated. No study investigated the sole effect of electronic self-monitoring of mood as an intervention in itself, but investigated the effect of different electronically delivered intervention programmes with electronic self-monitoring of mood represented as part of the intervention. The evidence of the effect of electronic self-monitoring was limited by methodological issues and by a lack of RCTs. Notably, three of the RCTs investigating the effect of electronically delivered interventions did not report on any researcher blinded outcomes [60, 61, 63] and thus introduces bias issues on the validity of the results from these studies and introducing the risk of overestimating or underestimating the beneficial and harmful effects of the interventions. One RCT [16], by the authors, reported on potentially harmful effects of electronic self-monitoring with more depressive symptoms but fewer manic symptoms in the intervention group. A paper by *Scott & Colom* discuss the issues of a differential effectiveness of psychological interventions for manic and depressive phases [77], and points out that the reasons for these differential effects are not clear. Manic prodromes are more distinct and may be easier to detect and treat more quickly and effectively with pharmacotherapy than depressive episodes [78]. On the contrary, depressive symptoms are more difficult to differentiate from normal day-to-day problems and may have a more gradual onset and prolonged duration [79]. Considering electronic self-monitoring a psychological intervention, the potential harmful effects on depressive symptoms as suggested by the findings from the RCT by the authors [16] highlight that electronic self-monitoring should not be uncritically used or implemented in clinical practice and that important aspects need further clarification before it is implemented as a standard tool.

If there would be an effect of electronic self-monitoring on the severity of depressive or manic symptoms, then self-monitoring would influence the variables it measures (mood). Whether that would be a threat to the reliability and validity is unknown and should be investigated further.

In westerns countries nearly everyone has at least one device that would be able to handle electronic self-monitoring. The use of computers and/or tablets as tools for electronic self-monitoring of mood require some technical skills by the user and can be quite expensive to acquire. However, most people have access to a computer or tablet and know how to interact with simple software systems. Computers and tablets allow for storage and visual presentation of self-monitored data making recognition of symptom patterns possible, thus potentially providing tools for increasing the patients’ illness insight and empowerment. PDAs represent a tool that is possible for the patients to carry with them during the day making continuous real-time electronic self-monitoring in naturalistic settings is possible. However, when using a separate and non-standard electronic device for self-monitoring of illness activity the risk of stigmatization is present [56]. Other electronic devices could replace PDAs as an electronic self-monitoring tool in the years to come. Unlike computers and PDAs, smartphones offer opportunities for continuous electronic self-monitoring in naturalistic environments that cannot be achieved using other types of IT platforms. Since most people carry their cell phone with them during most of the day and use it for communicative purposes through various platforms the risk of stigmatization is not present. Furthermore, the number of smartphone users worldwide has been estimated to reach 2.5 billion people by 2017 [80] which makes smartphones an obvious tool for electronic self-monitoring.

Patients willing to participate in studies using these kinds of novel and technical electronic interventions could represent a more motivated and technically oriented group of patients with higher degree of illness insight and willingness to use the electronic self-monitoring tools in question. As can be seen from some of the included articles, compliance to self-monitoring was highly variable, and patients participating were quite young. Elucidation of possible technical barriers for using an electronic device for self-monitoring among non-technically oriented patient groups, perhaps in patients with higher age than in the included studies, could be of interest. In addition, future studies should provide more information on excluded patients and reasons for declining to participate in studies in order to allow the readers to better assess the level of generalizability of the study results.

None of the included studies provided information regarding the economical part of the development and maintenance of the electronic self-monitoring IT platforms, and is likely a relevant factor in the development of future efforts in this area as well as for the clinical implementation.

### Limitations

Some limitations to the present review should be mentioned. Telemedicine in general and e-mental health are areas under great expansion and the investigations in this area are published in very diverse forms and places reflecting that this is an research area in the intersection between two areas of research- medical research and IT research. Therefore, conducting a search strategy that is able to capture all relevant scientific literature is a challenge. A search on Google Scholar alone on electronic self-monitoring in bipolar disorder resulted in 595.000 hits. Many commercial websites and smartphone applications (for both Android, iOS and Windows) for electronic self-monitoring exist in the App store and the Google Play store. The search strategy for the present review reflects that we aimed at systematically collecting and reviewing published scientific studies in order to get an overview of the validity of electronic mood self-monitoring compared to validated clinical rating scales and the evidence status related to using these kinds of electronic self-monitoring tools.

Most of the included studies had a daily and momentary frequency of self-monitoring, but one study used a frequency of self-monitoring on a weekly basis. A recent paper discussed differences in momentary and retrospective trait self-report techniques pointing out that retrospective self-monitoring is influenced by peak moments with greater salience of moments that occur closets in time to the assessment [13].

The interventions of the included RCTs were heterogeneous and often mood self-monitoring was a part of an intervention, which incorporated mood self-monitoring alongside other psychological interventions. In addition, the RCTS employed various monitoring IT platforms and included different clinically relevant outcomes. Thus, comparing not only different mood self-monitoring tools but also different interventions as well as outcomes is a big challenge.

It should be noted that the authors did not have access to the various electronic self-monitoring tools reviewed apart from one of the tools for smartphones [16, 59]. Further, as pointed out by others [44] all of the self-monitoring tools are different from one another allowing for calculation of many different measures of illness activity and also making it difficult to compare findings across studies.

One author (MFJ) selected all papers and extracted all data, and one of the co-authors (KM) independently checked the extracted data.

It should also be noted that in most of the articles the patients had to provide the hardware for the electronic self-monitoring themselves and no information regarding economical compensation was given. None of the included articles provided information regarding the cost of developing and maintaining the electronic software and the amount of possible technical problems with the electronic self-monitoring systems. These aspects are likely relevant factors that should be evaluated in future studies.

It would be interesting to investigate the validity between electronic mood self-monitored and validated clinical rating scales for depression and mania and the effect of electronic self-monitoring as an intervention in a non-technically oriented group of patients with bipolar disorder, and further to elucidate these aspects during full-blown affective episodes.

## Conclusions

This systematic review identified relatively few studies investigating IT platforms for electronic self-monitoring in adult patients with bipolar disorder. Electronic self-monitoring of mood in depression appears to be a valid measure of mood in contrast to self-monitoring of mood in mania. This calls for other new and more objective real-time electronic methods for monitoring the severity of manic symptoms.

The evidence of the effect of electronic self-monitoring tools investigated in RCTs was limited by methodological issues and, by small number of RCTs primarily investigating the effect of different electronically delivered intervention programmes. Crucially, the potential beneficial or harmful effect of electronic self-monitoring tools on clinically relevant researcher blinded outcomes has scarcely been investigated and is unknown. Whether electronic self-monitoring should be considered an instrument or as an intervention or both is unknown and future research should investigate this further.

There is a need for further research using rigorous methodology and more RCTs investigating the effect and economic consequences of electronic self-monitoring using different types of IT platforms in patients with bipolar disorder. Furthermore, future RCTs should elucidate possible harmful effects to inform whether the potential benefits are worth the costs and potential risks.

## Additional file

Additional file 1:
***Effect of electronic mood self-monitoring tools on clinically relevant outcomes in randomized controlled trials.*** (DOCX 24 kb)
