# Supplementary material for: Electronic self-monitoring of mood using IT platforms in adult patients with bipolar disorder: A systematic review of the validity and evidence
Source: BMC Psychiatry. 2016 Jan 15;16:7. doi: 10.1186/s12888-016-0713-0 (PMC4714425; doi:10.1186/s12888-016-0713-0)
Supplement: Additional file 1: — Effect of electronic mood self-monitoring tools on clinically relevant outcomes in randomized controlled trials. (DOCX 24 kb) [file 12888_2016_713_MOESM1_ESM.docx]

Appendix 1:

***Effect of electronic mood self-monitoring tools on clinically relevant outcomes in randomized controlled trials***

A total of seven RCTs were included in the systematic review. Further details on these RCTs including a total of 759 patients with bipolar disorder involving a follow-up period ranging from 12 weeks to 12 months [57, 58, 60–64] can also be found in Table 2.

Two of the RCTs aimed primarily at investigating differences in compliance rates between using an electronic mood self-monitoring tool compared to using paper-and-pencil mood self-monitoring [57, 58] and reported on differences in symptom scores as secondary outcomes. One study (n=48) reported on a RCT aimed at investigating the compliance to online mood self-monitoring (intervention group) compared to paper-and-pencil mood self-monitoring (control group) during a 90 days study period. Data on Clinical Global Impression Severity (CGI-S) score were also collected. The study reported that the intervention group showed significantly higher compliance to mood self-monitoring compared to the control group. No difference in CGI-S was found between the two groups [57]. Another study (n=40) reported on a RCT aimed at investigating the compliance to smartphone-based mood self-monitoring (intervention group) compared to paper-and-pencil mood self-monitoring (control group) during a 12 weeks study period. The study reported that the intervention group showed significantly higher variability of self-monitored mood compared to the control group. Further, a higher compliance to mood self-monitoring was found in the control group [58].

Five RCTs investigated the effect of different electronically delivered intervention programmes including a self-monitoring tool on symptoms of illness activity in patients with bipolar disorder [60–64]. One study (n=122) reported on a RCT investigating the effect of an online interactive recovery informed self-management intervention including self-monitoring (‘Living with Bipolar’) (intervention group) compared to treatment-as-usual (TAU) (and waiting list) (control group) during a six month study period. The intervention was based on principles of Cognitive Behavioral Therapy and psychoeducation and designed as a self-management intervention. The study reported that the intervention group showed significant improvement in self-assessed quality of life, recovery, symptoms severity and social functioning compared to the control group [60]. Another study (n=233) reported on a RCT investigating the effect of a web-based psychoeducational program including self-monitoring (Recovery Road for Bipolar Disorder) (intervention group) compared to a control group directed to web-sites on healthy lifestyles (and waiting list). The study reported that there were no significant differences between the two groups in any of the defined outcome (time to first self-reported recurrence, self-reported hospitalization and self-assessed disability) [61]. The third study (n=82) reported on a RCT investigating the effect of smartphone-based ecological momentary delivery of personalized self-management strategies based on self-reported mood scores (intervention group) compared to paper-and-pencil mood self-monitoring (control group). The study reported that post-treatment showed the intervention group significant greater reductions in clinically rated depressive symptoms, but not manic symptoms compared to the control group. The effect was not maintained at follow-up at the end of study [62]. The fourth study (n=156) reported on a RCT investigating the effect of an online program consisting of psychoeducation, mood tracking, discussion boards and cognitive behavioral therapy (MoodSwings Plus) (intervention group) compared to an online program consisting of psychoeducation, mood tracking and discussion boards (MoodSwings) (control group). The study reported that the intervention group showed significant reductions in self-assessed manic symptoms compared to the control group, but no difference was observed in relation to self-assessed depressive symptoms [63]. The last study, by the authors, (n=78) reported on a RCT investigating the effect of smartphone-based self-monitoring including a bi-directional feedback loop to the patients and clinicians (MONARCA) (intervention group) compared to receiving a placebo smartphone and TAU (control group). The study reported that there were no significant differences between the two groups in any of the defined outcomes (severity of depressive and manic symptoms, quality of life, self-assessed depressive and manic symptoms, coping strategies, cognitive function, and perceived stress). Sub-analyses showed that the intervention group had more depressive symptoms and fewer manic symptoms than the control group [64].
